# Nurses’ Knowledge and Practices Regarding Delirium Management in Intensive Care Units in Cyprus: A Cross-Sectional Study

**DOI:** 10.3390/healthcare14081039

**Published:** 2026-04-14

**Authors:** Evanthia Asimakopoulou, Kyriakos Alexandrou, Maria Foka, Anna Vavlitou, Petroula M. Mavrikiou

**Affiliations:** 1 Department of Nursing, Frederick University, 7 Y. Frederickou Str, 1036 Nicosia, Cyprus; kyriakosalexa@gmail.com; 2 Intensive Care Forum, 2252 Nicosia, Cyprus; fokamaria1@gmail.com; 3 Intensive Care Unit, Nicosia General Hospital, 2029 Nicosia, Cyprus; a.vavlitou@shso.org.cy; 4 Department of Business Administration, Frederick University, 1036 Nicosia, Cyprus; p.mavrikiou@frederick.ac.cy

**Keywords:** delirium, intensive care, nurses, patient safety, sedation, knowledge, critical care, Cyprus

## Abstract

**Highlights:**

**What are the main findings?**

- ICU nurses demonstrated moderate–high knowledge of delirium.
- Routine delirium screening was reported by only 27.1% of nurses.

**What are the implications of the main findings?**

- Structured delirium education and training programs are needed to improve nurses’ competencies in delirium assessment and prevention.
- Implementation of standardized screening tools and protocols may strengthen ICU patient safety.

**Abstract:**

Background: Delirium is a frequent and serious complication in intensive care units (ICUs), associated with increased mortality, prolonged mechanical ventilation, extended length of stay, and long-term cognitive impairment. This study aimed to assess ICU nurses’ knowledge and practices regarding delirium management in Cyprus and to identify predictors of knowledge. Methods: A cross-sectional study was conducted among nurses working in adult ICUs in Cyprus. Data were collected using a structured self-administered questionnaire that included demographic characteristics, sedation and analgesia practices, and an adapted Delirium Knowledge Questionnaire incorporating ICU-specific items. Results: A total of 70 ICU nurses participated, most of whom were female (60%) with a mean ICU experience of 5.1 years. Only 27.1% reported daily delirium screening, although 65.2% perceived delirium as frequent. Sedation protocols were reported by 34.3%, sedation scales were used by 44.3%, and daily sedation interruption by 61.4%. Only 15.7% had received formal delirium training, while 87.1% expressed the need for further education. Knowledge scores were moderate to high (68.5–84.0%), with higher scores among nurses with prior training and female nurses (*p* = 0.003). Hospital type was associated with sedation practices, with greater use of sedation scales in public ICUs (*p* < 0.001) and propofol more commonly used as first-line sedation compared with midazolam in private ICUs (*p* = 0.018). Conclusions: Although ICU nurses demonstrated moderate knowledge of delirium, systematic screening and protocolized management remain suboptimal. Structured education and standardized implementation strategies are required to strengthen patient safety in critical care settings.

## 1. Introduction

The intensive care unit (ICU) represents a high-risk clinical environment in which patient safety is continuously challenged by the complexity of critical illness and the intensity of therapeutic interventions. Despite advances in evidence-based critical care [1,2,3], preventable harm remains substantial, with the World Health Organization (WHO) estimating that up to 10% of hospitalized patients experience adverse events, the majority of which are preventable [4]. Within this context, neurological complications—particularly delirium—have emerged as central determinants of both short- and long-term patient outcomes.

In critical care, delirium is no longer conceptualized solely as an acute neuropsychiatric syndrome, characterized by disturbances in attention, awareness, and cognition [5,6], but as a manifestation of acute brain dysfunction within the broader framework of organ failure [7,8]. Recent research increasingly position ICU delirium at the interface of psychiatry, neurology, and critical care medicine, reflecting complex pathophysiological processes including neuroinflammation, neurotransmitter imbalance, and disrupted cerebral perfusion [9,10]. This reconceptualization underscores delirium as a form of acute encephalopathy with direct implications for long-term neurological integrity rather than a reversible consequence of illness. Moreover, delirium is not just a temporary complication of critical illness but an important factor contributing to long-term cognitive decline in ICU survivors, affecting recovery and quality of life [7]. Accumulating evidence demonstrates that delirium is a key driver of post-intensive care syndrome (PICS), particularly its cognitive domain. Longitudinal studies indicate that survivors of critical illness frequently develop persistent cognitive impairment affecting memory, executive function, and attention, with trajectories comparable to mild traumatic brain injury or early dementia [11].

The incidence of delirium in ICU populations remains high, affecting approximately 25–80% of patients [9,10,12], particularly those requiring mechanical ventilation [9,13]. Despite its high prevalence and established clinical significance, delirium remains substantially under-recognized in critical care settings [14]. Failure to detect and manage delirium early contributes to prolonged exposure to modifiable risk factors such as deep sedation, immobility, and sleep disruption, all of which are implicated in adverse neurocognitive trajectories [15]. From a patient safety perspective, delirium is strongly associated with increased mortality, prolonged mechanical ventilation, extended ICU and hospital length of stay, and higher rates of institutionalization [15]. Mortality risk among ICU patients with delirium is estimated to be two to four times higher compared to non-delirious patients, while six-month mortality also significantly increases [16]. Furthermore, delirium imposes a substantial economic burden on healthcare systems, with annual costs reaching billions of dollars globally. These clinical and economic repercussions position delirium as a critical patient safety issue, particularly within high-risk environments such as ICUs [17].

Patient safety in critical care settings depends on early detection of clinical deterioration, implementation of evidence-based protocols, and effective interprofessional collaboration [18]. The 2018 PADIS guidelines emphasize routine delirium assessment using validated tools, risk factor modification, and non-pharmacological preventive strategies [19]. However, implementation of these recommendations remains inconsistent worldwide, largely due to gaps in knowledge, limited training, and variability in clinical practice [20,21]. Within this framework, frontline healthcare providers—particularly nurses—play a pivotal role [22]. Continuous bedside presence places nurses at the center of delirium detection, monitoring, and intervention. Their ability to identify early cognitive and behavioral changes, implement validated screening tools such as CAM-ICU, and apply non-pharmacological prevention strategies directly influences both the incidence and duration of delirium. Evidence suggests that structured, nurse-driven interventions, including components of the ABCDEF bundle, are associated with reduced delirium burden and improved long-term outcomes [23,24].

Conversely, inadequate training and inconsistent assessment practices contribute to missed diagnoses and delayed interventions, perpetuating the cycle of acute brain dysfunction and subsequent cognitive decline [25]. Evidence from multiple countries suggests that nurses’ knowledge and practices regarding delirium management remain suboptimal. Studies in Iraq [26], the Netherlands [20], China [21], and Poland [27] report moderate to low knowledge levels and inconsistent screening practices. Barriers identified: shortage of personnel, interprofessional communication gaps, and difficult-to-assess patients [28]. Evidence indicates that targeted educational interventions significantly enhance nurses’ recognition skills, protocol adherence, and overall patient safety, highlighting the need for systematic evaluation of delirium-related competencies [29,30].

In Cyprus, evidence regarding ICU nurses’ knowledge and practices related to delirium remains limited. Understanding these factors is essential for identifying system-level gaps and designing targeted interventions aimed at improving both patient safety and long-term neurocognitive outcomes [31]. Therefore, the present study aims to assess ICU nurses’ knowledge and practices regarding delirium management and to explore factors associated with knowledge variability within the Cypriot critical care context.

## 2. Materials and Methods

### 2.1. Study Design

A cross-sectional study was conducted to assess ICU nurses’ knowledge and practices regarding delirium management in Cyprus. A cross-sectional design was considered appropriate to provide a comprehensive snapshot of current knowledge levels and clinical practices related to delirium screening, prevention, and sedation management. This approach aligns with similar international studies examining delirium-related competencies and patient safety practices among critical care nurses [31,32].

### 2.2. Setting and Participants

The study was conducted in five adult ICUs located in Nicosia and Limassol, Cyprus (two public, two private ICUs within the General Healthcare System (GHS) and one private non-GHS ICU), and included general ICUs managing medical, surgical, and mixed critically ill adult patients.

Eligible participants were registered nurses who:

1. Held a nursing degree (Bachelor’s or higher);
2. Provided direct bedside care to adult ICU patients;
3. Had at least six months of ICU clinical experience;
4. Gave informed consent to participate.

Nurses in administrative roles or those not directly involved in patient care were excluded.

A convenience sampling approach was employed. Eligible nurses were recruited based on their availability during scheduled shifts in participating ICUs. Following institutional approval, the research team approached nurses who met the inclusion criteria and invited them to participate. Participation was voluntary, and all eligible nurses present during the data collection period had an equal opportunity to participate. This approach was considered appropriate given the operational constraints of ICU settings, including shift work patterns and workload demands [33]. No formal a priori power calculation was conducted, as the study was designed as an exploratory cross-sectional investigation. Therefore, the sample size was not intended to detect statistically powered differences between subgroups but to provide an initial assessment of ICU nurses’ knowledge and practices in the Cypriot context.

### 2.3. Data Collection Procedure

Data collection was conducted over a three-month period (October–December 2025). After obtaining institutional permissions, eligible nurses were invited to participate during scheduled shifts. Participants received an information sheet explaining the study purpose, confidentiality assurances, and voluntary participation. Written informed consent was obtained prior to questionnaire completion. The sample size was determined based on feasibility and consistency with similar cross-sectional studies conducted in critical care nursing populations. Due to the absence of prior national data on ICU nurses’ knowledge of delirium in Cyprus, a formal power calculation was not feasible. A total of 70 participants were included, representing approximately 15% of the estimated national ICU nursing workforce. This sample size is considered adequate for exploratory analyses and is comparable to similar studies assessing delirium-related knowledge and practices among ICU nurses internationally [32]. The response rate was 70%, which is considered acceptable in survey-based healthcare research. The studies involving healthcare professionals often report response rates between 40% and 70% due to workload and shift patterns [20,21,33]. It should be noted that the limited representation of private non-GHS ICUs reflects the structure of the Cypriot healthcare system, where the majority of private hospitals are integrated into the General Healthcare System (GHS), and only a small number of private non-GHS units operate nationally.

### 2.4. Instruments

Data were collected using a structured, anonymous self-administered questionnaire developed for the Cypriot ICU context. The questionnaire consisted of two main parts:

Part 1: Demographic and professional characteristics

A structured questionnaire was developed to collect demographic and professional characteristics, which were collected using structured closed-ended questions, including nominal variables (gender, ICU specialization, hospital type), ordinal variables (educational level), and continuous variables (age, years of nursing experience, and years of ICU experience).

Part 2: ICU Sedation–Analgesia–Delirium Management Questionnaire

This section assessed:

- ICU sedation and analgesia management practices, including: presence of written sedation protocols, use of sedation scales, daily sedation interruption practices, frequency of use of sedative agents and analgesic use patterns. Responses were recorded using categorical or Likert-type scales.
- Knowledge, attitudes, and practices regarding Delirium, including: daily delirium screening practices, use of validated tools, perceived importance of prevention, previous delirium-related training and Delirium knowledge assessment:

The questionnaire was developed based on the Delirium Knowledge Questionnaire (DKQ), originally designed and developed by Hare et al. [34] and later refined by Detroyer et al. [29], to assess nurses’ knowledge of delirium across key domains, including clinical features, risk factors, and management strategies. In addition, 10 ICU-specific items were developed by the research team to reflect critical care-related aspects of delirium, including mechanical ventilation, sedation practices, circadian rhythm disruption, and early mobilization. Item generation was informed by current evidence and international guidelines, including the PADIS recommendations.

All items were structured as dichotomous (True/False) questions. Correct responses were scored as 1 and incorrect responses as 0. Domain-specific scores and a total knowledge score were calculated by summing item responses, with higher scores indicating greater knowledge.

As no validated Greek version of the DKQ was available, the instrument was translated and culturally adapted for the Cypriot ICU context. Initial translation was performed using existing Greek-language delirium-related literature [35,36] and direct adaptation from the original English version. The translated items were subsequently reviewed by two bilingual experts in critical care nursing to ensure semantic equivalence, clarity, and contextual relevance. Minor modifications were made to improve wording and ensure alignment with local clinical terminology. Although a formal forward–backward translation process was not conducted, efforts were made to preserve the conceptual meaning of all items.

### 2.5. Validity and Reliability

Content validity was assessed through expert evaluation by a panel consisting of academic faculty in critical care nursing and experienced ICU clinicians. Experts reviewed each item for relevance, clarity, and representativeness of the construct of delirium knowledge in ICU settings. Feedback focused on item wording, clinical applicability, and comprehensiveness of the domains. Minor revisions were made to improve clarity and contextual appropriateness. Overall, the instrument was considered to have satisfactory content validity for use in this study.

A pilot study was conducted with 10 ICU nurses from a hospital not included in the main sample to assess clarity, feasibility, and completion time. Participants provided feedback on item comprehension and questionnaire structure. Based on this feedback, minor wording adjustments were made to improve clarity and readability. No substantial changes to item content or structure were required. Pilot data were not included in the final analysis.

The internal consistency of the DKQ-35 was acceptable (Cronbach’s α = 0.775). The ICU-specific subscale demonstrated lower internal consistency (α = 0.663), which is considered borderline acceptable. This finding may be attributed to the relatively small number of items and the multidimensional nature of the construct, as the subscale includes diverse aspects of ICU-related delirium knowledge. Similar variability in internal consistency has been reported in exploratory instruments assessing complex clinical knowledge domains. Therefore, the reliability results should be interpreted with caution.

### 2.6. Data Analysis

Data were analyzed using the IBM Statistical Package for the Social Sciences (SPSS, IBM, Armonk, NY, USA), v.29. Descriptive statistics were used to summarize demographic characteristics and knowledge and recognition scores (mean, standard deviation, frequency, and percentage). Inferential statistics included:

- Independent samples *t*-tests to compare delirium knowledge scores across groups (e.g., gender, education level, ICU experience categories).
- Chi-square tests to examine associations between categorical variables (e.g., hospital type and sedation practices).
- Correlation analysis (Pearson correlation) to explore relationships between continuous variables such as age, years of ICU experience, and knowledge scores.

ICU experience was categorized into two groups (1–4 years vs. ≥5 years) to reflect differences in clinical experience. This threshold is supported by nursing theory and empirical evidence, suggesting that progression toward higher levels of clinical proficiency and clinical reasoning occurs with increasing years of experience, particularly beyond the early years of practice [37].

Statistical significance was set at *p* < 0.05.

### 2.7. Ethical Considerations

The study was conducted in accordance with the Declaration of Helsinki, and approved by the Cyprus National Bioethics Committee (Approval No: ΕΕΒΚ/ΕΠ/2025/96, date: 8 July 2025) and by the participating hospitals’ administrations. Participation was voluntary, anonymity was ensured, and no identifiable personal data were collected. Participants were informed that they could withdraw at any time without consequences.

### 2.8. AI-Assisted Technologies Statement

An AI-assisted language tool (ChatGPT—5.3, OpenAI, San Francisco, CA, USA) was used solely for English language editing and clarity improvement. All scientific content and final manuscript decisions were made by the authors.

## 3. Results

### 3.1. Demographic Characteristics of Participants

A total of 70 ICU nurses were included in the study. Participants were predominantly female (60.0%), with a mean age of 31.8 years (SD = 6.0). Most held a bachelor’s degree (70.0%), while 30.0% had completed postgraduate studies. Only 10.0% reported formal ICU specialization. The mean years of professional nursing experience was 8.4 (SD = 5.7), including 5.1 years (SD = 4.1) of ICU experience. The majority were employed in private hospitals within the GHS (60.0%). The demographic characteristics are summarized in Table 1.

### 3.2. Sedation and Analgesia Practices

A written sedation or sedation–analgesia protocol was reported by 34.3% of participants. Familiarity with international sedation guidelines was generally limited, with 58.6% reporting little or no familiarity. Sedation scales were used by 44.3% of respondents, with Richmond Agitation-Sedation Scale (RASS) being the most frequently reported tool. Daily sedation interruption was applied by 61.4% of ICU nurses. Propofol was the predominant sedative agent, with 92.9% reporting very frequent use, and 77.1% identifying it as their first-line agent. Midazolam was also widely used, whereas thiopental was less frequently administered. Dexmedetomidine demonstrated moderate variability in use. Sedative agents were commonly administered in combination with analgesics. Regarding analgesics, remifentanil (62.9%) and fentanyl (52.9%) were most frequently used, followed by morphine. Ketamine was rarely administered. Continuous neuromuscular blockade beyond short-term use was predominantly reported as occasional (55.7%).

### 3.3. Delirium Screening and Education

Only 27.1% of participants reported performing daily delirium screening. Among those who screened routinely *(n* = 10), CAM-ICU was used by six nurses, while four reported using Nu-DESC. Despite limited screening implementation, 65.2% perceived delirium as a frequent occurrence in their ICU. Prevention of delirium was considered highly important, with 87.2% rating it as very or extremely important. However, only 15.7% had received formal training on delirium management. The majority (87.1%) expressed the need for further education, with face-to-face seminars being the preferred educational format (51.4%). The distribution of sedation, analgesia, and delirium management practices is summarized in Table 2.

### 3.4. Delirium Knowledge

The knowledge levels of ICU nurses regarding delirium are presented in Table 3. Overall, moderate to high knowledge scores were observed across all domains of the Delirium Knowledge Questionnaire (DKQ), with some variability between sections.

In Part A (clinical features and symptomatology; 10 items), the mean score was 7.56 (SD = 1.68, median = 8, IQR = 3) out of 10, corresponding to 75.6% of the maximum possible score. Scores ranged from 4 to 10, with 9 nurses (12.9%) achieving the maximum score. In Part B (risk factors and causes; 11 items), the mean score was 7.53 (SD = 1.36, median = 8, IQR = 1) out of 11, corresponding to 68.5% of the maximum possible score. Observed scores ranged from 4 to 10. This section demonstrated the lowest relative percentage of maximum score among all domains and the smallest standard deviation (SD = 1.36), indicating comparatively lower variability in responses. In Part C (prevention and management; 14 items), the mean score was 10.63 (SD = 1.80, median = 11, IQR = 2) out of 14, corresponding to 75.9% of the maximum possible score. Scores ranged from 5 to 14, with 2 participants achieving full marks. This domain exhibited the largest dispersion (SD = 1.80), suggesting greater variability in knowledge related to prevention and management strategies.

The ICU-specific section (DKQ-ICU; 10 items) demonstrated the highest relative performance, with a mean score of 8.40 (SD = 1.43, median = 9, IQR = 1), corresponding to 84.0% of the maximum possible score. Scores ranged from 4 to 10, with 16 participants (22.9%) achieving the maximum score. Variability in this section was moderate (SD = 1.43), falling between Parts B and C.

Comparatively, the highest performance was observed in the ICU-specific domain, followed by Parts C and A, while Part B demonstrated the lowest relative performance. In terms of dispersion, knowledge variability was greatest in Part C and lowest in Part B.

### 3.5. Predictors of Delirium Knowledge Among ICU Nurses

Independent samples *t*-tests were conducted to examine differences in overall delirium knowledge scores according to selected demographic and professional characteristics. Nurses who had previously attended delirium-related training demonstrated significantly higher knowledge scores compared with those without such training (37.55 ± 2.91 vs. 33.47 ± 4.26, *p* = 0.003). Gender differences were also observed, with female nurses demonstrating significantly higher delirium knowledge scores compared with male nurses (35.33 ± 3.79 vs. 32.29 ± 4.49, *p* = 0.003). In contrast, no significant differences in overall knowledge scores were observed according to hospital type (33.86 ± 4.36 vs. 34.22 ± 4.35, *p* = 0.747) or educational level (34.62 ± 4.64 vs. 33.90 ± 4.21, *p* = 0.526). Additionally, when ICU experience was categorized into two groups (1–4 years vs. ≥5 years), nurses with ≥5 years of ICU experience demonstrated significantly higher scores in the prevention domain of the questionnaire compared with those with 1–4 years of experience (10.00 ± 1.98 vs. 11.11 ± 1.52, *p* = 0.011) (Table 4). No significant differences were observed in the other knowledge domains.

Chi-square tests were performed to examine associations between categorical variables related to ICU practices and professional characteristics. Significant associations were identified between hospital type and sedation-related clinical practices (Table 5). The use of sedation scales was reported significantly more frequently in public ICUs compared with private ICUs (90.5% vs. 24.5%, *p* < 0.001). Similarly, hospital type was significantly associated with the choice of first-line sedative agent, with propofol more commonly reported in public ICUs and midazolam more frequently reported in private ICUs (95.2% vs. 69.4% and 4.8% vs. 30.6%, *p* = 0.018). No statistically significant associations were observed between hospital type and the presence of written sedation protocols or the implementation of daily sedation interruption. Additionally, no association was identified between gender and educational level.

Correlation analyses were conducted to examine the relationship between continuous variables and overall delirium knowledge scores. Neither years of ICU experience (*p* = 0.746) nor age (*p* = 0.622) were significantly associated with overall knowledge scores. Furthermore, ICU experience did not differ significantly between male and female nurses when analyzed as a continuous variable (*p* = 0.315).

A multiple linear regression analysis was conducted (Table 6) to further examine the independent predictors of total delirium knowledge. The results confirmed that only gender and delirium-related training were statistically significant predictors of knowledge. Specifically, gender demonstrated a positive association with knowledge scores (β = 3.493, *p* < 0.001), indicating that female nurses had higher knowledge levels compared to male nurses. Delirium-related training showed a negative association (β = −4.674, *p* < 0.001), assuming that higher values correspond to lower educational attainment, the result suggests that nurses with higher education demonstrated greater knowledge. Both variables remained statistically significant (*p* < 0.001), indicating their independent contribution to delirium knowledge.

## 4. Discussion

Within the patient safety framework, delirium represents a preventable and modifiable complication when early detection and multicomponent interventions are implemented. In the present study, delirium prevention was perceived as highly important, with 87.2% of participants rating it as very or extremely important. This finding aligns with previous studies reporting high awareness of delirium but inconsistent implementation of screening practices [38]. However, this awareness does not consistently translate into clinical practice [39]. Despite the high perceived importance observed in our study, the proportion of nurses reporting routine daily delirium screening was notably low (27.1%). Although 65.2% perceived delirium as frequent in their ICU, routine structured assessment using validated tools such as CAM-ICU or Nu-DESC was inconsistently implemented. This discrepancy highlights a knowledge–practice gap, which has been reported internationally [20,21]. Failure to systematically assess delirium may delay recognition of hypoactive presentations, a commonly underdiagnosed subtype associated with poor outcomes [40].

Sedation practices in this study demonstrated partial alignment with current recommendations. The predominant use of propofol and the reported application of daily sedation interruption by 61.4% of nurses are consistent with evidence favoring light sedation strategies [1]. However, only one-third (34.3%) reported the presence of a written sedation protocol, and familiarity with international guidelines was limited. The use of structured protocols and standardized assessment tools is associated with reduced delirium incidence and improved outcomes [2,41]. Limited protocol use may contribute to practice variability and increased risk of preventable harm [42]. Studies from countries such as Greece, Poland, and the Netherlands have identified comparable barriers, including limited training, absence of standardized protocols, and variability in clinical practice [20,22,31]. The relatively low implementation of sedation protocols observed in this study may indicate additional structural limitations at the institutional level, particularly in smaller healthcare systems. Addressing these gaps requires not only educational interventions but also system-level strategies, including the implementation of standardized protocols, integration of delirium assessment tools into routine workflows, and strengthening of guideline dissemination and adherence.

Overall delirium knowledge levels were moderate to high, with total domain scores ranging from 68.5% to 84.0%. The highest performance was observed in the ICU-specific section (84%), suggesting that nurses may be more familiar with context-specific aspects of delirium. Conversely, knowledge related to risk factors and causes (Part B) demonstrated the lowest relative performance (68.5%). Knowledge of risk factors is essential for prevention and risk stratification [1,43]. Lower performance in this domain may partially explain inconsistencies in screening and prevention practices. Knowledge of clinical features alone may not translate into proactive risk mitigation. This may result in delayed recognition and suboptimal interventions [31]. Numerous studies highlight this deficit, indicating that many intensive care unit nurses lack comprehensive knowledge regarding delirium, its assessment, and management [31,44,45].

A key finding of this study is that prior delirium-related training was a significant predictor of knowledge scores. This finding aligns with previous international studies demonstrating that targeted educational interventions significantly improve delirium recognition and management competencies [26,32,46]. Clinical experience may also contribute to improved understanding of prevention strategies. Previous research has similarly highlighted the role of clinical experience in enhancing nurses’ competence in delirium management, particularly in areas related to prevention and non-pharmacological interventions [31,32]. In a recent study, prior delirium training was the strongest predictor of both knowledge and positive attitudes [47]. The high proportion of nurses expressing the need for further education (87.1%) indicates awareness of knowledge gaps and readiness for professional development. From a systems perspective, this represents an opportunity for institutional quality improvement initiatives.

A statistically significant difference in knowledge scores between male and female nurses was observed in this study. Although previous research suggests that female nurses may report greater engagement in continuing education and evidence-based practice activities [48,49], the factors contributing to this difference were not specifically examined in the current study and should be interpreted with caution.

Hospital type was associated with sedation practices. Sedation scales were used more frequently in public ICUs compared with private ICUs. The use of validated tools such as the RASS is recommended in international guidelines [1]. Hospital type was also associated with the choice of first-line sedative agent, with propofol more commonly reported in public ICUs and midazolam more frequently used in private ICUs (*p* = 0.018). This variability may reflect differences in institutional protocols and prescribing practices. Current guidelines recommend propofol or dexmedetomidine over benzodiazepines due to the increased risk of delirium associated with agents such as midazolam [1], and similar inter-hospital variability in sedation practices has been reported previously [2,41].

A key finding is the coexistence of moderate-to-high knowledge levels with low rates of systematic screening, well documented also internationally [20,21,50]. Barriers may include workload pressures, lack of institutional protocols, insufficient interdisciplinary coordination, absence of performance monitoring and delirium care difficulties [37]. These findings indicate that knowledge alone is insufficient, and structured implementation strategies are required to translate evidence into practice [2]. Embedding delirium monitoring within electronic health records, integrating sedation and delirium checklists into routine workflow, and strengthening interprofessional collaboration may enhance translation of knowledge into practice.

This study has some limitations that should be considered when interpreting the findings. First, although the knowledge assessment was based on the Delirium Knowledge Questionnaire (DKQ), a formally validated Greek-language version specifically tailored to ICU nurses in Cyprus is not currently available. The instrument used in this study was linguistically adapted and reviewed for content validity; however, a full cross-cultural validation process was not conducted. Additionally, the internal consistency of the ICU-specific subscale was borderline acceptable and these results related to this subscale should be interpreted with caution. Second, the cross-sectional design precludes causal inferences regarding the relationship between knowledge levels and clinical practices. Finally, the use of self-reported data may be subject to response bias, potentially overestimating adherence to recommended practices.

Future research should focus on formal psychometric validation of a Greek-language delirium knowledge instrument and on interventional studies evaluating the impact of structured education on patient safety outcomes in critical care.

## 5. Conclusions

In conclusion, this study demonstrates that although ICU nurses in Cyprus exhibit moderate to high knowledge regarding delirium, systematic screening and protocolized management remain limited. Prior training significantly enhances knowledge, underscoring the importance of structured educational initiatives. Bridging the knowledge–practice gap through evidence-based protocols, continuous training, and organizational support is essential for strengthening patient safety in critical care environments. From a patient safety perspective, delirium prevention and early detection represent modifiable targets within ICU systems. Given that delirium is associated with increased mortality and long-term cognitive decline, enhancing nurses’ competence in delirium management has important clinical implications beyond the ICU stay. While not directly assessed in this study, improved delirium management may contribute to better long-term patient outcomes and more efficient healthcare delivery. In smaller healthcare systems such as Cyprus, efforts toward standardization of delirium assessment practices could further strengthen patient safety.

## Figures and Tables

**Table 1:** Demographic characteristics of ICU nurses (*n* = 70).

Categorical Variables	*n* (%)
Variable Name	
**Gender**	
Male	28 (40.0)
Female	42 (60.0)
**Education Level**	
Bachelor’s Degree	49 (70.0)
Master’s Degree (MSc)	21 (30.0)
**ICU Specialization**	
Yes	7 (10.0)
No	63 (90.0)
**Type of Hospital**	
Public	21 (30.0)
Private (GHS)	42 (60.0)
Private (non-GHS)	7 (10.0)
**Continuous Variables**	**Mean (SD)**
**Variable Name**	
Age (years)	31.79 (6.00)
Years of Nursing Experience	8.40 (5.74)
Years of ICU Experience	5.10 (4.15)

**Table 2:** Sedation, Analgesia, and Delirium practices among ICU nurses (*n* = 70).

Variable	*n* (%)
**Sedation Practices**	
Written sedation protocol	24 (34.3)
Use of sedation scale	31 (44.3)
Daily sedation interruption	43 (61.4)
First-line sedative: Propofol	54 (77.1)
Propofol (very frequent use)	65 (92.9)
Remifentanil (very frequent use)	44 (62.9)
Fentanyl (very frequent use)	37 (52.9)
Continuous NMBA (occasional use)	39 (55.7)
**Delirium Screening & Education**	
Daily delirium screening	19 (27.1)
Perceived delirium as frequent	45 (65.2)
Received formal training	11 (15.7)
Need for further education	61 (87.1)
Preferred format: Face-to-face seminar	36 (51.4)

**Table 3:** Domain-level analysis of Delirium knowledge scores.

Domain	Items (*n*)	Score Range	Mean (SD)	Median (IQR)	% οf Maximum Score
**Part A** **Symptoms**	10	0–10	7.56 (1.68)	8 (3)	75.6%
**Part B** **Risk Factors**	11	0–11	7.53 (1.36)	8 (1)	68.5%
**Part C** **Prevention**	14	0–14	10.63 (1.80)	11 (2)	75.9%
**DKQ-ICU**	10	0–10	8.40 (1.43)	9 (1)	84.0%

**Table 4:** Delirium knowledge domain scores according to ICU experience (1–4 years vs. ≥5 years).

Domain	Mean (1–4 yrs) SD	Mean (≥5 yrs) SD	*p*-Value
**Part A** **Symptoms**	7.61 (1.72)	7.43 (1.70)	0.681
**Part B** **Risk Factors**	7.47 (1.27)	7.57 (1.52)	0.784
**Part C** **Prevention**	10.00 (1.98)	11.11 (1.52)	0.011 *
**DKQ-ICU**	8.68 (1.07)	8.07 (1.76)	0.078

* *p* < 0.05.

**Table 5:** Association between hospital type and sedation practices in ICU nurses.

Variable	Public Hospitals (%)	Private Hospitals ^†^ (%)	*p*-Value
Sedation scale use	90.5	24.5	<0.001 **
Daily sedation interruption	76.2	55.1	0.097
Propofol first line	95.2	69.4	0.018
Midazolam first line	4.8	30.6	

^†^ GHS and non-GHS; ** *p* < 0.001.

**Table 6:** Multivariable Analysis of Predictors of Delirium Knowledge.

Variable	Unstandardized Coefficients B	*t*-Statistic	Sig. Value	Tolerance	VIF
Constant	37.139	14.358	0.000		
Gender	3.493	3.803	0.000	0.984	1.017
Delirium Training	−4.674	−3.780	0.000	0.984	1.017

Dependent Variable: Delirium Training. Independent Variables: Gender (1: man, 2: woman), Training (1: Received training, 2: Not received training).

## Data Availability

The data presented in this study are available on request from the corresponding author, due to ethical and privacy restrictions, as the dataset contains sensitive personal and clinical information and participant consent does not allow for public sharing.

## Abbreviations

The following abbreviations are used in this manuscript:

CAM-ICU	Confusion Assessment Method for the ICU
DKQ	Delirium Knowledge Questionnaire
GHS	General Healthcare System
ICU	Intensive Care Unit
Nu-DESC	Nursing Delirium Screening Scale
PADIS	Pain, Agitation/Sedation, Delirium, Immobility, and Sleep Disruption
RASS	Richmond Agitation Sedation Scale
WHO	World Health Organization

## References

[1] Devlin J.W., Skrobik Y., Gélinas C., Needham D.M., Slooter A.J.C., Pandharipande P.P., Watson P.L., Weinhouse G.L., Nunnally M.E., Rochwerg B. (2018). Clinical practice guidelines for the prevention and management of pain, agitation/sedation, delirium, immobility, and sleep disruption in adult patients in the ICU. Crit. Care Med..

[2] Pun B.T., Balas M.C., Barnes-Daly M.A., Thompson J.L., Aldrich J.M., Barr J., Byrum D., Carson S.S., Devlin J.W., Engel H.J. (2019). Caring for Critically Ill Patients with the ABCDEF Bundle: Results of the ICU Liberation Collaborative in Over 15,000 Adults. Crit. Care Med..

[3] Asimakopoulou E., Theodosis-Nobelos P., Triantis C. (2018). Pain assessment and management in intensive care unit: Pharmacological and non-pharmacological approaches. Hell. J. Nurs..

[4] World Health Organization (WHO) (2021). Global Patient Safety Action Plan 2021–2030: Towards Eliminating Avoidable Harm in Health Care.

[5] American Psychiatric Association (2013). Diagnostic and Statistical Manual of Mental Disorders.

[6] Brummel N.E., Girard T.D. (2019). Delirium in the critically ill patient. Handb. Clin. Neurol..

[7] Ramnarain D., Pouwels S., Fernández-Gonzalo S., Navarra-Ventura G., Balanzá-Martínez V. (2023). Delirium-related psychiatric and neurocognitive impairment and the association with post-intensive care syndrome-A narrative review. Acta Psychiatr. Scand..

[8] Slooter A.J.C., Otte W.M., Devlin J.W., Arora R.C., Bleck T.P., Claassen J., Duprey M.S., Ely E.W., Kaplan P.W., Latronico N. (2020). Updated nomenclature of delirium and acute encephalopathy. Intensive Care Med..

[9] Stollings J.L., Kotfis K., Chanques G., Pun B.T., Pandharipande P.P., Ely E.W. (2021). Delirium in critical illness: Clinical manifestations, outcomes, and management. Intensive Care Med..

[10] Slooter A.J., Van De Leur R.R., Zaal I.J. (2017). Delirium in critically ill patients. Handb. Clin. Neurol..

[11] Fiest K.M., Soo A., Hee Lee C., Niven D.J., Ely E.W., Doig C.J., Stelfox H.T. (2021). Long-Term Outcomes in ICU Patients with Delirium: A Population-based Cohort Study. Am. J. Respir. Crit. Care Med..

[12] Arumugam S., El-Menyar A., Al-Hassani A., Strandvik G., Asim M., Mekkodithal A., Mudali I., Al-Thani H. (2017). Delirium in the Intensive Care Unit. J. Emerg. Trauma Shock.

[13] Collet M.O., Caballero J., Sonneville R., Bozza F.A., Nydahl P., Schandl A., Wøien H., Citerio G., van den Boogaard M., Hästbacka J. (2018). AID-ICU cohort study co-authors. Prevalence and risk factors related to haloperidol use for delirium in adult intensive care patients: The multinational AID-ICU inception cohort study. Intensive Care Med..

[14] Marjanovic S., Berisavac I., Tutus V., Boskovic S., Omcikus M., Jankovic T., Hadzibegovic A., Ratkovic S., Opacic J., Stanisavljevic J. (2025). Delirium Management in Critical Care: Are We Moving Forward or Still Treading Water?. Med. Sci..

[15] Rowley-Conwy G. (2018). Barriers to delirium assessment in the intensive care unit: A literature review. Intensive Crit. Care Nurs..

[16] Ely E.W., Shintani A., Truman B., Speroff T., Gordon S.M., Harrell F.E., Inouye S.K., Bernard G.R., Dittus R.S. (2004). Delirium as a predictor of mortality in mechanically ventilated patients in the intensive care unit. JAMA.

[17] Sasidharan S., Dhillon H. (2025). Shadows in the Mind: Navigating Intensive Care Unit Delirium and Prevention. APIK J. Intern. Med..

[18] Asimakopoulou E., Theodosis-Nobelos P., Triantis C. (2024). Advances in the management of posttraumatic stress disorder after critical illness: A narrative review. Emerg. Crit. Care Med..

[19] Park S.Y., Lee H.B. (2019). Prevention and management of delirium in critically ill adult patients in the intensive care unit: A review based on the 2018 PADIS guidelines. Acute Crit. Care.

[20] Trogrlić Z., Ista E., Ponssen H.H., Schoonderbeek J.F., Schreiner F., Verbrugge S.J., Dijkstra A., Bakker J., van der Jagt M. (2017). Attitudes, knowledge and practices concerning delirium: A survey among intensive care unit professionals. Nurs. Crit. Care.

[21] Xing H., Zhu S., Liu S., Xia M., Jing M., Dong G., Ni W., Li L. (2022). Knowledge, attitudes and practices of ICU nurses regarding subsyndromal delirium among 20 hospitals in China: A descriptive cross-sectional survey. BMJ Open.

[22] Parissopoulos S., Timmins F., Mpouzika M., Mantzorou M., Kapadochos T., Papagaroufali E. (2023). Intensive Care Nurses’ Experience of Caring in Greece; A Qualitative Study. Healthcare.

[23] Asimakopoulou E., Madianos M. (2015). Posttraumatic Stress Disorder After Discharge From Intensive Care Units in Greater Athens Area. J. Trauma Nurs..

[24] Brooke J., Manneh C. (2018). Caring for a patient with delirium in an acute hospital: The lived experience of cardiology, elderly care, renal, and respiratory nurses. Int. J. Nurs. Pract..

[25] Blevins C.S., DeGennaro R. (2018). Educational Intervention to Improve Delirium Recognition by Nurses. Am. J. Crit. Care.

[26] Najafi Ghezeljeh T., Jaber Muhaibes F., Haghani S., Kifah Mubdir A. (2023). Nurses’ knowledge and ability to diagnose delirium in intensive care units of Iraq teaching hospitals. J. Client-Centered Nurs. Care.

[27] Krupa S., Friganović A., Oomen B., Benko S., Mędrzycka-Dąbrowska W. (2022). Nurses’ Knowledge about Delirium in the Group of Intensive Care Units Patients. Int. J. Environ. Res. Public Health.

[28] Byrnes T., Gombar M., Price S., Cochran A., Love K., Gregory A., Rankin V., Daye-Whitehead K. (2024). Delirium is Under-Detected on Routine Screening with the CAM: A Sub-Study from World Delirium Awareness Day. Delirium.

[29] Detroyer E., Dobbels F., Debonnaire D., Irving K., Teodorczuk A., Fick D.M., Joosten E., Milisen K. (2016). The effect of an interactive delirium e-learning tool on healthcare workers’ delirium recognition, knowledge and strain in caring for delirious patients: A pilot pre-test/post-test study. BMC Med. Educ..

[30] Connell J.M., Duggan M.C., Wilson J.E. (2023). Why We Must Prevent and Appropriately Manage Delirium. AMA J. Ethics.

[31] Lange S., Mȩdrzycka-Da Browska W., Tomaszek L., Wujtewicz M., Krupa S. (2023). Nurses’ knowledge, barriers and practice in the care of patients with delirium in the intensive care unit in Poland-A cross-sectional study. Front. Public Health.

[32] Dai D., Tian Y., Jing W., Liu H., Xu Y. (2025). Knowledge, attitude, and practice of intensive care nurses toward delirium: A multicenter cross-section study. Front. Med..

[33] Polit F.D., Beck C.T. (2021). Nursing Research: Generating and Assessing Evidence for Nursing Practice.

[34] Hare M., Wynaden D., McGowan S., Landsborough I., Speed G. (2008). A questionnaire to determine nurses’ knowledge of delirium and its risk factors. Contemp. Nurse.

[35] Ouzouni C., Nakakis K. (2012). Nurses’ knowledge of care for patients with delirium. Rostrum Asclepius.

[36] Papastavrou E., Papaioannou M., Evripidou M., Tsangari H., Kouta C., Merkouris A. (2019). Development of a Tool for the Assessment of Nurses’ Attitudes Toward Delirium. J. Nurs. Meas..

[37] Dong J., Sui W., Gong X., Wang L., Ni Q., Yan R., Yi J., Ding Y., Zhuang Y. (2026). Impact of Nurses’ Knowledge, Self-Efficacy and Clinical Reasoning Competency on Difficulties in Caring for Patients with Delirium in the Intensive Care Unit: A Cross-Sectional Study. J. Clin. Nurs..

[38] Glynn L., Corry M. (2015). Intensive care nurses’ opinions and current practice in relation to delirium in the intensive care setting. Intensive Crit. Care Nurs..

[39] Zamoscik K., Godbold R., Freeman P.J.I., Nursing C.C. (2017). Intensive care nurses’ experiences and perceptions of delirium and delirium care. Intensive Crit. Care Nurs..

[40] Meagher D. (2009). Motor subtypes of delirium: Past, present and future. Int. Rev. Psychiatry.

[41] Skrobik Y., Ahern S., Leblanc M., Marquis F., Awissi D.K., Kavanagh B.P. (2010). Protocolized intensive care unit management of analgesia, sedation, and delirium improves analgesia and subsyndromal delirium rates. Anesth. Analg..

[42] Pandharipande P., Ely E.W. (2006). Sedative and analgesic medications: Risk factors for delirium and sleep disturbances in the critically ill. Crit. Care Clin..

[43] Zaal I.J., Devlin J.W., Peelen L.M., Slooter A.J. (2015). A systematic review of risk factors for delirium in the ICU. Crit. Care Med..

[44] Elliott S.R. (2014). ICU delirium: A survey into nursing and medical staff knowledge of current practices and perceived barriers towards ICU delirium in the intensive care unit. Intensive Crit. Care Nurs..

[45] Zhou W., Zheng Q., Huang M., Zhang C., Zhang H., Yang L., Wu T., Gan X. (2023). Knowledge, attitude, and practice toward delirium and subtype assessment among Chinese clinical nurses and determinant factors: A multicentre cross-section study. Front. Psychiatry.

[46] Chang Y.-L., Hsieh M.-J., Chang Y.-C., Yeh S.-L., Chen S.-W., Tsai Y.-F. (2023). Self-Efficacy of Caring for Patients in the Intensive Care Unit with Delirium: Development and Validation of a Scale for Intensive Care Unit Nurses. Aust. Crit. Care.

[47] Shibu S., Manoj Kumar S., Yashpal S., Ajay Kumar S., Harpreet D. (2026). Healthcare Professionals’ Knowledge, Attitudes, and Practices Regarding Delirium in Intensive Care Units: A Cross-sectional Study. Curr. Med. Issues.

[48] Aiken L.H., Cimiotti J.P., Sloane D.M., Smith H.L., Flynn L., Neff D.F. (2011). Effects of nurse staffing and nurse education on patient deaths in hospitals with different nurse work environments. Med. Care.

[49] Ozturk Z., Kaya M., Aksoy M., Özlü Z.K. (2025). Investigation of the factors affecting delirium evaluation by intensive care nurses: A cross-sectional descriptive study. BMC Nurs..

[50] Devlin J.W., Fong J.J., Howard E.P., Skrobik Y., McCoy N., Yasuda C., Marshall J. (2008). Assessment of delirium in the intensive care unit: Nursing practices and perceptions. Am. J. Crit. Care.

